# 

**Published:** 1872-02

**Authors:** 


### OHIO DENTAL COLLEGE ASSOCIATION.

The annual meeting of this body will be held at the College on Tuesday, March 6th, at 10 o'clock A. M., at which time it is very important that all the members be present, as work of great interest to the Association, the College and the profession is to be transacted. Let all come with a strong purpose to assist in doing work that shall result in building up this institution, which, in a sense that no other does, belongs to the profession—especially to the profession of the West.

### MISSISSIPPI VALLEY DENTAL SOCIETY.

The twenty-eighth annual meeting of the Mississippi Valley Dental Society will be held in the Hall of the Ohio Dental College, beginning on Wednesday, March 6th, prox., at 10 o'clock A. M., and continue three days.

A general and cordial invitation is extended to all to be present.

### ORDER OF BUSINESS.

1st. Calling and correcting the roll.

2d. Reading the minutes of the last session.

3d. Reports of Standing Committees—Executive Committee—Committee on Membership—Committee on Ethics.

4th. Reports of Special Committees.

5th. Election of members and payment of dues.

### ESSAYS AND DISCUSSIONS.

SUBJECTS:—1st. What are the legitimate requirements and expectations of the dentist, in view of the necessities that exist, and the advantages available?

2d. What are the obstacles in the way of a complete fulfillment of professional responsibility, and how are they to be overcome?

3d. What are the duties of the dentist to his profession?

4th. What is the present status of the profession in ability to *protect*, *rescue* and *save* the natural teeth?

5th. What are the means requisite for securing the largest measure of success in the preservation of the teeth?

6th. What is the present status of the profession in ability to supplement the loss of the natural teeth, and the defects occasioned thereby; and what are the impediments in the way of higher attainments in this direction?

7th. What means shall be employed for extending the sphere of usefulness of the dentist?

All papers pertaining to any of the above questions to be read upon the introduction of the subject.

It is desirable that each member select and elaborate some particular thought in connection with each of the questions; that the entire ground may be occupied, and the discussions may be full and concise, and that the different members may not travel over the same ground. There should be a paper upon each subject.

The election of officers will take place at 11 o'clock A. M., of the third day.

Your committee would recommend that one or two general quizzes be held during the sessions.

Dr. Corydon Palmer will be present, and give some of his unparalleled illustrations and demonstrations.

J. TAFT,<br>

W. H. SHADOAN, } *Ex. Com.*<br>

E. C. SLOAN,

### CONTENTS.

COMMUNICATIONS.

Dental Jottings .......... 53
Therapeutical Action of Arsenic .......... 61

PROCEEDINGS OF SOCIETIES.

Discussion before the Sixth Annual Meeting of the Ohio State Dental Society .......... 65

EDITORIAL.

Old Colony Dental Society .......... 88

A Visit—Educational .......... 88

Still at School .......... 91

"Monsieur Tonsin Come Again"—Rubber Subject .......... 92

Celluloid Base .......... 94

Ohio Dental College Association .......... 94

Mississippi Valley Dental Society .......... 95

---

### The Dental Register,

### OF THE WEST.

A Monthly Magazine, devoted entirely to the interest of the Dental Profession.

**Terms $3.00 per Year. Single Nos. 25 Cents.**

EDITED BY PROF. J. TAFT,

And Published by SPENCER & MOORE of the Ohio Dental Depot.

The volume commences in January and closes in December.

The Register being issued monthly is always fresh, therefore indispensable to the practitioner who desires to keep posted up with the new inventions and improvements which are daily developed. Neither expense nor effort will be spared to give value and variety to its contents. Articles will be illustrated with well executed engravings whenever they will add to the interest or assist in explanation.

Its extensive circulation and frequency of issue makes it one of the BEST DENTAL ADVERTISING MEDIUMS in the United States.

RATES OF ADVERTISING.

One page, one year, $50. Half page, one year, $30. Quarter page, one year, $20.

All advertising bills payable in advance, or at furthest after the issue of the first number containing the advertisement. All transient advertisements to be paid for in advance.

☞CONTRIBUTIONS SOLICITED.

Exclusively Editorial Communications should be addressed to

**Dr. J. TAFT, Editor.**

The latest number of the Register may be ordered with or without other goods at any time, and all letters should be addressed to DENTAL REGISTER,

Or **SPENCER & MOORE,**

***Room No. 1 Pike's Opera House, Cincinnati, O.***

